# Spliceosome malfunction causes neurodevelopmental disorders with overlapping features

**DOI:** 10.1172/JCI171235

**Published:** 2024-01-02

**Authors:** Dong Li, Qin Wang, Allan Bayat, Mark R. Battig, Yijing Zhou, Daniëlle G.M. Bosch, Gijs van Haaften, Leslie Granger, Andrea K. Petersen, Luis A. Pérez-Jurado, Gemma Aznar-Laín, Anushree Aneja, Miroslava Hancarova, Sarka Bendova, Martin Schwarz, Radka Kremlikova Pourova, Zdenek Sedlacek, Beth A. Keena, Michael E. March, Cuiping Hou, Nora O’Connor, Elizabeth J. Bhoj, Margaret H. Harr, Gabrielle Lemire, Kym M. Boycott, Meghan Towne, Megan Li, Mark Tarnopolsky, Lauren Brady, Michael J. Parker, Hanna Faghfoury, Lea Kristin Parsley, Emanuele Agolini, Maria Lisa Dentici, Antonio Novelli, Meredith Wright, Rachel Palmquist, Khanh Lai, Marcello Scala, Pasquale Striano, Michele Iacomino, Federico Zara, Annina Cooper, Timothy J. Maarup, Melissa Byler, Robert Roger Lebel, Tugce B. Balci, Raymond Louie, Michael Lyons, Jessica Douglas, Catherine Nowak, Alexandra Afenjar, Juliane Hoyer, Boris Keren, Saskia M. Maas, Mahdi M. Motazacker, Julian A. Martinez-Agosto, Ahna M. Rabani, Elizabeth M. McCormick, Marni J. Falk, Sarah M. Ruggiero, Ingo Helbig, Rikke S. Møller, Lino Tessarollo, Francesco Tomassoni Ardori, Mary Ellen Palko, Tzung-Chien Hsieh, Peter M. Krawitz, Mythily Ganapathi, Bruce D. Gelb, Vaidehi Jobanputra, Ashley Wilson, John Greally, Sébastien Jacquemont, Khadijé Jizi, Ange-Line Bruel, Chloé Quelin, Vinod K. Misra, Erika Chick, Corrado Romano, Donatella Greco, Alessia Arena, Manuela Morleo, Vincenzo Nigro, Rie Seyama, Yuri Uchiyama, Naomichi Matsumoto, Ryoji Taira, Katsuya Tashiro, Yasunari Sakai, Gökhan Yigit, Bernd Wollnik, Michael Wagner, Barbara Kutsche, Anna C.E. Hurst, Michelle L. Thompson, Ryan Schmidt, Linda Randolph, Rebecca C. Spillmann, Vandana Shashi, Edward J. Higginbotham, Dawn Cordeiro, Amanda Carnevale, Gregory Costain, Tayyaba Khan, Benoît Funalot, Frederic Tran Mau-Them, Luis Fernandez Garcia Moya, Sixto García-Miñaúr, Matthew Osmond, Lauren Chad, Nada Quercia, Diana Carrasco, Chumei Li, Amarilis Sanchez-Valle, Meghan Kelley, Mathilde Nizon, Brynjar O. Jensson, Patrick Sulem, Kari Stefansson, Svetlana Gorokhova, Tiffany Busa, Marlène Rio, Hamza Hadj Habdallah, Marion Lesieur-Sebellin, Jeanne Amiel, Véronique Pingault, Sandra Mercier, Marie Vincent, Christophe Philippe, Clemence Fatus-Fauconnier, Kathryn Friend, Rebecca K. Halligan, Sunita Biswas, Jane Rosser, Cheryl Shoubridge, Mark Corbett, Christopher Barnett, Jozef Gecz, Kathleen Leppig, Anne Slavotinek, Carlo Marcelis, Rolph Pfundt, Bert B.A. de Vries, Marjon A. van Slegtenhorst, Alice S. Brooks, Benjamin Cogne, Thomas Rambaud, Zeynep Tümer, Elaine H. Zackai, Naiara Akizu, Yuanquan Song, Hakon Hakonarson

**Affiliations:** 1 Center for Applied Genomics, and; 2 Division of Human Genetics, The Children’s Hospital of Philadelphia, Philadelphia, Pennsylvania, USA.; 3 Department of Pediatrics, University of Pennsylvania Perelman School of Medicine, Philadelphia, Pennsylvania, USA.; 4 Raymond G. Perelman Center for Cellular and Molecular Therapeutics, Children’s Hospital of Philadelphia, Philadelphia, Pennsylvania, USA.; 5 Department of Regional Health Research, University of Southern Denmark, Odense, Denmark.; 6 Department for Epilepsy Genetics and Personalized Medicine, Danish Epilepsy Centre, Dianalund, Denmark.; 7 Department of Drug Design and Pharmacology, University of Copenhagen, Copenhagen, Denmark.; 8 Department of Clinical Genetics, Erasmus Medical Center, Rotterdam, The Netherlands.; 9 Department of Genetics, Center for Molecular Medicine, University Medical Center Utrecht, Utrecht University, Utrecht, The Netherlands.; 10 Department of Genetics and Metabolism, Randall Children’s Hospital at Legacy Emanuel Medical Center, Portland, Oregon, USA.; 11 Centro de Investigación Biomédica en Red de Enfermedades Raras, ISCIII, Madrid, Spain.; 12 Genetic Service, Hospital del Mar Research Institute (IMIM), Barcelona, Spain.; 13 Universitat Pompeu Fabra, Barcelona, Spain.; 14 Pediatric Neurology, Hospital del Mar Research Institute (IMIM), Barcelona, Spain.; 15 Department of Biology and Medical Genetics, Charles University Second Faculty of Medicine and University Hospital Motol, Prague, Czech Republic.; 16 Children’s Hospital of Eastern Ontario Research Institute, University of Ottawa, Ottawa, Ontario, Canada.; 17 Ambry Genetics, Aliso Viejo, California, USA.; 18 Invitae, San Francisco, California, USA.; 19 Division of Neuromuscular and Neurometabolic Disorders, Department of Paediatrics, McMaster University Children’s Hospital, Hamilton, Ontario, Canada.; 20 Department of Clinical Genetics, Sheffield Children’s Hospital, Sheffield, United Kingdom.; 21 University Health Network, Toronto, Ontario, Canada.; 22 University of Illinois College of Medicine, Mercy Health Systems, Rockford, Illinois, USA.; 23 Laboratory of Medical Genetics, Translational Cytogenomics Research Unit, Bambino Gesù Children’s Hospital, IRCCS, Rome, Italy.; 24 Medical Genetics Unit, Academic Department of Pediatrics, IRCCS, Ospedale Pediatrico Bambino Gesù, Rome, Italy.; 25 Genetics and Rare Diseases Research Division, Ospedale Pediatrico Bambino Gesù, IRCCS, Rome, Italy.; 26 Rady Children’s Institute for Genomic Medicine, San Diego, California, USA.; 27 Division of Pediatric Neurology, Department of Pediatrics, University of Utah School of Medicine, Salt Lake City, Utah, USA.; 28 Division of Pediatric Pulmonary and Sleep Medicine, University of Utah, Salt Lake City, Utah, USA.; 29 Department of Neurosciences, Rehabilitation, Ophthalmology, Genetics, Maternal and Child Health, Università Degli Studi di Genova, Genoa, Italy.; 30 Pediatric Neurology and Muscular Diseases Unit, and; 31 Medical Genetics Unit, IRCCS, Istituto Giannina Gaslini, Genoa, Italy.; 32 Department of Genetics, Southern California Permanente Medical Group, Kaiser Permanente, San Diego, California, USA.; 33 Department of Genetics, Kaiser Permanente, Los Angeles, California, USA.; 34 Center for Development, Behavior and Genetics, SUNY Upstate Medical University, Syracuse, New York, USA.; 35 Division of Genetics, Department of Paediatrics, London Health Sciences Centre, London, Ontario, Canada.; 36 Greenwood Genetic Center, Greenwood, South Carolina, USA.; 37 Division of Genetics and Genomics, Boston Children’s Hospital, Boston, Massachusetts, USA.; 38 Division of Genetics and Metabolism, Mass General Hospital for Children, Boston, Massachusetts, USA.; 39 APHP. SU, Reference Center for Intellectual Disabilities Caused by Rare Causes, Department of Genetics and Medical Embryology, Hôpital Trousseau, Paris, France.; 40 Institute of Human Genetics, Friedrich-Alexander-Universität Erlangen-Nürnberg, Erlangen, Germany.; 41 Department of Genetics, Hospital Pitié-Salpêtrière, Paris, France.; 42 Department of Human Genetics, Academic Medical Center, and; 43 Laboratory of Genome Diagnostics, Department of Human Genetics, Amsterdam UMC, University of Amsterdam, Amsterdam, The Netherlands.; 44 Division of Medical Genetics, Department of Pediatrics, UCLA, Los Angeles, California, USA.; 45 Mitochondrial Medicine Frontier Program, Division of Human Genetics, Department of Pediatrics,; 46 Division of Neurology, and; 47 The Epilepsy NeuroGenetics Initiative (ENGIN), Children’s Hospital of Philadelphia, Philadelphia, Pennsylvania, USA.; 48 Department of Neurology, University of Pennsylvania, Philadelphia, Pennsylvania, USA.; 49 Department of Biomedical and Health Informatics (DBHi), Children’s Hospital of Philadelphia, Philadelphia, Pennsylvania, USA.; 50 Department of Epilepsy Genetics and Personalized Medicine, Danish Epilepsy Centre, Dianalund, Denmark.; 51 Mouse Cancer Genetics Program, Center for Cancer Research, National Cancer Institute (NCI), Frederick, Maryland, USA.; 52 Institute for Genomic Statistics and Bioinformatics, University Hospital Bonn, Rheinische Friedrich-Wilhelms-Universität Bonn, Bonn, Germany.; 53 New York Genome Center, New York, New York, USA.; 54 Department of Pathology, Columbia University Irving Medical Center, New York, New York, USA.; 55 Mindich Child Health and Development Institute and the Departments of Pediatrics and Genetics and Genomic Sciences, Icahn School of Medicine, New York, New York, USA.; 56 Department of Genetics, Albert Einstein College of Medicine, Bronx, New York, USA.; 57 Division of Genetics and Genomics, CHU Ste-Justine Hospital and CHU Sainte-Justine Research Centre, University of Montreal, Montreal, Quebec, Canada.; 58 Division of Genetics and Genomics, CHU Ste-Justine Hospital and CHU Sainte-Justine Research Centre, University of Montreal, Montreal, Quebec, Canada.; 59 INSERM UMR 1231, Genetics of Developmental Anomalies, Université de Bourgogne Franche-Comté, Dijon, France.; 60 UF Innovation en Diagnostic Génomique des Maladies Rares, CHU Dijon Bourgogne, Dijon, France.; 61 FHU-TRANSLAD, Fédération Hospitalo-Universitaire Translational Medicine in Developmental Anomalies, CHU Dijon Bourgogne, Dijon, France.; 62 Medical Genetics Department, Centre de Référence Maladies Rares CLAD-Ouest, CHU Hôpital Sud, Rennes, France.; 63 Division of Genetic, Genomic, and Metabolic Disorders, Children’s Hospital of Michigan, Detroit, Michigan, USA.; 64 Central Michigan University College of Medicine, Discipline of Pediatrics, Mount Pleasant, Michigan, USA.; 65 Research Unit of Rare Diseases and Neurodevelopmental Disorders, Oasi Research Institute-IRCCS, Troina, Italy.; 66 Medical Genetics, Department of Biomedical and Biotechnological Sciences, University of Catania, Catania, Italy.; 67 Oasi Research Institute-IRCCS, Troina, Italy.; 68 Telethon Institute of Genetics and Medicine (TIGEM), Pozzuoli, Naples, Italy.; 69 Department of Precision Medicine, University of Campania “Luigi Vanvitelli”, Naples, Italy.; 70 Department of Human Genetics, Yokohama City University Graduate School of Medicine, Yokohama, Japan.; 71 Department of Obstetrics and Gynecology, Juntendo University, Tokyo, Japan.; 72 Department of Rare Disease Genomics, Yokohama City University Hospital, Yokohama, Japan.; 73 Department of Pediatrics, Graduate School of Medical Sciences, Kyushu University, Fukuoka, Japan.; 74 Department of Pediatrics, Karatsu Red Cross Hospital, Saga, Japan.; 75 Institute of Human Genetics, University Medical Center Göttingen, Göttingen, Germany.; 76 DZHK (German Center for Cardiovascular Research), partner site Göttingen, Göttingen, Germany.; 77 Cluster of Excellence “Multiscale Bioimaging: from Molecular Machines to Networks of Excitable Cells” (MBExC), University of Göttingen, Göttingen, Germany.; 78 Kinderzentrum Oldenburg, Sozialpädiatrisches Zentrum, Diakonisches Werk Oldenburg, Oldenburg, Germany.; 79 Department of Genetics, University of Alabama at Birmingham, Birmingham, Alabama, USA.; 80 HudsonAlpha Institute for Biotechnology, Huntsville, Alabama, USA.; 81 Department of Pathology and Laboratory Medicine, Children’s Hospital Los Angeles, Los Angeles, California, USA.; 82 Keck School of Medicine of the University of Southern California, Los Angeles, California, USA.; 83 Division of Medical Genetics, Children’s Hospital Los Angeles, California, USA.; 84 Department of Pediatrics–Medical Genetics, Duke University School of Medicine, Durham, North Carolina, USA.; 85 Genome Diagnostics, Department of Paediatric Laboratory Medicine, and; 86 Division of Clinical and Metabolic Genetics, The Hospital for Sick Children, Toronto, Ontario, Canada.; 87 Department of Genetics, Hôpital Henri-Mondor APHP and CHI Creteil, University Paris Est Creteil, IMRB, Inserm U.955, Creteil, France.; 88 Institute of Medical and Molecular Genetics (INGEMM), Hospital Universitario La Paz, Madrid, Spain.; 89 Department of Pediatrics, Division of Clinical and Metabolic Genetics, The Hospital for Sick Children, University of Toronto, Toronto, Ontario, Canada.; 90 Department of Genetic Counselling, Division of Clinical and Metabolic Genetics, Hospital for Sick Children, Ottawa, Ontario, Canada.; 91 Department of Molecular Genetics, University of Toronto, Toronto, Ontario, Canada.; 92 Department of Clinical Genetics, Cook Children’s Hospital, Fort Worth, Texas, USA.; 93 Division of Genetics, Department of Paediatrics, McMaster University, Hamilton, Ontario, Canada.; 94 Division of Genetics and Metabolism, Department of Pediatrics, University of South Florida, Tampa, Florida, USA.; 95 Nantes Université, CHU Nantes, Medical Genetics Department, Nantes, France.; 96 Nantes Université, CNRS, INSERM, l’Institut du Thorax, Nantes, France.; 97 deCODE genetics/Amgen Inc., Reykjavik, Iceland.; 98 Faculty of Medicine, School of Health Sciences, University of Iceland, Reykjavik, Iceland.; 99 Aix Marseille University, Inserm, U1251-MMG, Marseille Medical Genetics, Marseille, France.; 100 Department of Medical Genetics, Timone Hospital, APHM, Marseille, France.; 101 Department of Genomic Medicine of Rare Disorders, Necker Hospital, APHP Center, University Paris Cité, Paris, France.; 102 Rare Disease Genetics Department, APHP, Hôpital Necker, Paris, France.; 103 Université Paris Cité, Inserm, Institut Imagine, Embryology and Genetics of Malformations Laboratory, Paris, France.; 104 Laboratoire de Biologie Médicale Multi-Sites SeqOIA (laboratoire-seqoia.fr), Paris, France.; 105 Reference Center for Hereditary Metabolic Diseases, CHU Dijon Bourgogne, Dijon, France.; 106 Genetics and Molecular Pathology, SA Pathology, Adelaide, South Australia, Australia.; 107 Metabolic Clinic, and; 108 Department of General Medicine, Women’s and Children’s Hospital, Adelaide, South Australia, Australia.; 109 Adelaide Medical School and Robinson Research Institute, The University of Adelaide, South Australia, Australia.; 110 Pediatric and Reproductive Genetics Unit, Women’s and Children’s Hospital, North Adelaide, South Australia, Australia.; 111 South Australian Health and Medical Research Institute, Adelaide, South Australia, Australia.; 112 Genetic Services, Kaiser Permenante of Washington, Seattle, Washington, USA.; 113 Department of Pediatrics, Cincinnati Children’s Hospital Medical Center, Cincinnati, Ohio, USA.; 114 Department of Human Genetics, Donders Institute for Brain, Cognition and Behaviour, Radboud University Medical Center, Nijmegen, The Netherlands.; 115 Kennedy Center, Department of Clinical Genetics, Copenhagen University Hospital, Rigshospitalet, Glostrup, Denmark.; 116 Department of Clinical Medicine, University of Copenhagen, Copenhagen, Denmark.; 117 Department of Pathology and Laboratory Medicine, University of Pennsylvania, Philadelphia, Pennsylvania, USA.

**Keywords:** Development, Genetics, Genetic diseases, Neurodevelopment, iPS cells

## Abstract

Pre-mRNA splicing is a highly coordinated process. While its dysregulation has been linked to neurological deficits, our understanding of the underlying molecular and cellular mechanisms remains limited. We implicated pathogenic variants in *U2AF2* and *PRPF19*, encoding spliceosome subunits in neurodevelopmental disorders (NDDs), by identifying 46 unrelated individuals with 23 de novo *U2AF2* missense variants (including 7 recurrent variants in 30 individuals) and 6 individuals with de novo *PRPF19* variants. Eight *U2AF2* variants dysregulated splicing of a model substrate. Neuritogenesis was reduced in human neurons differentiated from human pluripotent stem cells carrying two *U2AF2* hyper-recurrent variants. Neural loss of function (LoF) of the *Drosophila* orthologs *U2af50* and *Prp19* led to lethality, abnormal mushroom body (MB) patterning, and social deficits, which were differentially rescued by wild-type and mutant *U2AF2* or *PRPF19*. Transcriptome profiling revealed splicing substrates or effectors (including Rbfox1, a third splicing factor), which rescued MB defects in *U2af50*-deficient flies. Upon reanalysis of negative clinical exomes followed by data sharing, we further identified 6 patients with NDD who carried *RBFOX1* missense variants which, by in vitro testing, showed LoF. Our study implicates 3 splicing factors as NDD-causative genes and establishes a genetic network with hierarchy underlying human brain development and function.

## Abstract 2

Spliceosome defects involving U2AF2, PRPF19, and RBFOX1 variants contribute to neurodevelopmental disorders by disrupting neuritogenesis and brain development.

## Graphical abstract

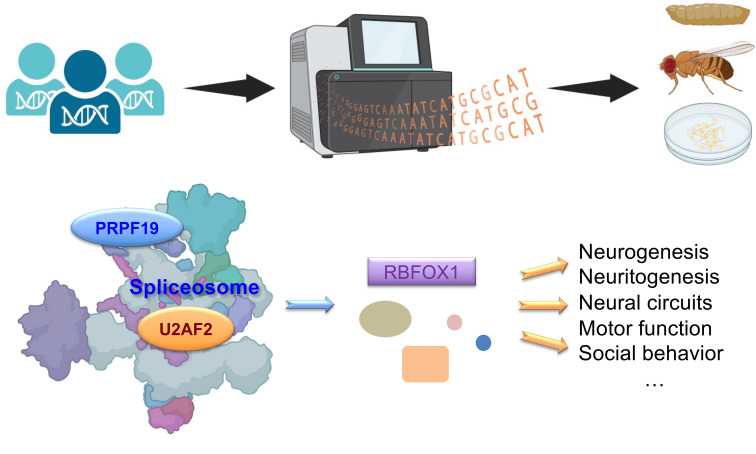

## Introduction

Neurodevelopmental disorders (NDDs) are a heterogeneous group of neurological and related neuropsychiatric conditions including intellectual disability (ID), autism spectrum disorder (ASD), and developmental and epileptic encephalopathies that manifest during childhood (1–3). Over the past 2 decades, there has been an impressive expansion with respect to gene discoveries for NDDs, revealing more than 1,500 genes in different signaling pathways (3, 4), including many transcriptional regulators such as DNA/histone modifiers (5–7) and chromatin-regulatory protein complexes (8–11). De novo variants are increasingly appreciated as playing a substantial causal role in the development of these disorders, and clinical laboratories now routinely report de novo variants in candidate genes not definitively associated with NDD (2, 12), which further facilitates the gene discovery effort. Over the past decade, a genetic diagnosis was obtained, on average, in one-third of individuals with NDD, which is a fundamental step forward in offering biological insights into underlying molecular mechanisms and providing individuals who have an undiagnosed disorder with a prognosis, counseling for recurrence risk, and precision medicine considerations. However, a mechanistic understanding of how these NDD-associated genes are networked and how their malfunction leads to neurodevelopmental or cognitive impairments are major unanswered questions.

Most protein-coding genes in humans are transcribed as pre-mRNAs that contain a series of exons and introns. Following transcription, the removal of introns during the process of pre-mRNA splicing is required before the nascent transcript is translated into a protein. Alternative splicing is a highly coordinated and precise process that involves *cis*-acting exonic and intronic elements (i.e., consensus motif sequences) and numerous *trans* pre-mRNA–binding protein factors, including heterogeneous nuclear ribonucleoproteins (hnRNPs), uridine-rich small nuclear RNPs (snRNPs), the PRP19 complex (also known as the nineteen complex [NTC)]), and multiple RNA-binding proteins (RBPs) (13–15). It is widely acknowledged that aberrant splicing of many NDD-related genes due to mutations or dysregulation of *cis-* or *trans*-acting elements/factors has profound influences on neuronal differentiation, neuronal patterning, and synaptic function (16–18), however, germline variants in core spliceosome components have rarely been implicated in NDDs (19–23).

Here, using integration of clinical phenotyping, exome/genome sequencing, protein structure analysis, modeling in flies and human pluripotent stem cells (hPSCs), and transcriptomics, we mapped the genetic architecture of 3 NDD-associated genes, determined their roles in neurodevelopment and behavior, and validated the pathogenicity of these human variants. We first identified 46 and 6 individuals with undiagnosed disorders, who harbored mostly de novo heterozygous variants in U2 small nuclear RNA auxiliary factor 2 (*U2AF2*), and pre-mRNA processing factor 19 (*PRPF19*), respectively, which encode spliceosomal subunits. We demonstrated that these pathogenic variants lead to converging neurodevelopmental phenotypes, including, but not limited to, developmental delay, ID, and autism. Transcriptome profiling of fly brains deficient in their orthologs *U2af50* and *Prp19*, hPSCs with CRISPR/Cas9 knockin, and patient-derived induced pluripotent stem cells (iPSCs) uncovered potential splicing substrates and downstream effectors, including RNA-binding Fox protein 1 (*Rbfox1*), which encodes an RBP that recognizes specific intronic sequences (UGCAUG and GCAUG) and regulates neuronal alternative splicing (24, 25). Overexpression of the downstream effectors partially rescued the neural defects in *U2af50*-deficient flies, establishing a genetic network of NDD-associated susceptibilities with hierarchy. Importantly, we subsequently identified 6 individuals carrying de novo missense variants in *RBFOX1*, who exhibited similar neurodevelopmental features. Therefore, by combining fly and human genetics, our work reveals a mechanistic network of NDD-associated genes involved in the spliceosome machinery and implicated in phenotypically overlapping disorders and identifies an evolutionarily conserved genetic hotspot for orchestrating neurodevelopment and function.

## Results

### U2AF2 variant carriers have a neurodevelopmental phenotype.

Phenotype and genotype data from 46 unrelated individuals with an NDD and a heterozygous missense variant in *U2AF2* (OMIM 191318) were ascertained through GeneMatcher and Matchermaker Exchange (26, 27). The cohort consisted of 27 female and 19 male probands spanning from 6 months to 24 years of age at the time of data collection, including 1 individual (individual 3) who was recruited from the Deciphering Developmental Disorders (DDD) study (www.ddduk.org) and 1 (individual 27) who was described elsewhere (28).

The affected individuals had substantial overlapping features, which are summarized in Table 1 and described in more detail in Supplemental Table 1 (supplemental material available online with this article; https://doi.org/10.1172/JCI171235DS1). All individuals except 2 (individuals 27 and 33, who died before 6 months of age) had developmental delay ranging from mild to severe, and most of them had global developmental delay involving speech and language, cognition, motor skills, and social behavior. ID was present in 30 of 34 (88%) of the individuals assessed, with a severity ranging from mild (*n* = 22) to severe (*n* = 8) ID. Four study participants (individuals 7, 11, 30, and 36) had borderline ID, with an IQ ranging from 74 to 79. Of the 10 remaining individuals, 7 were too young to perform formal IQ testing, and contact was lost with 3 individuals. Various neurological features were observed in the cohort. Hypotonia was identified in 25 of 37 probands (68%). Seizures occurred in 23 of 41 participants (56%), 15 of whom had febrile seizures (65%). Brain MRI was performed in 34 probands, and 14 (41%) had abnormal imaging findings, including delayed myelination, ventricle asymmetry, atrophy of the cerebral hemisphere, absent olfactory bulbs and tracts, dysmorphic inferior frontal gyri, and, the most prevalent finding, a thin or poorly developed corpus collosum (9 of 14 individuals). A formal diagnosis of ASD was made in 6 individuals, with 5 additional individuals having autistic features (11 of 39), and a number of different behavioral disturbances (i.e., anxiety, attention deficit disorder [ADD], attention deficit hyperactivity disorder [ADHD], aggression, obsession, stubbornness, tics, etc.) were observed in 22 of 39 individuals (56%), with a total of 64% (25 of 39) of the participants identified as having behavioral issues. Ten individuals had postnatal short stature (height ≤ –2 SD; 10 of 40), 5 individuals had microcephaly (head circumference [HC] ≤ –2 SD), 3 individuals had macrocephaly (HC ≥ +2 SD), and 4 individuals had obesity. Vision abnormality was a prominent feature in this cohort (20 of 37), and hearing loss was less pervasive (7 of 38). In 11 of 42 individuals (26%), heart abnormalities were reported. In 27 of 37 individuals, additional congenital abnormalities were observed and involved skeletal defects affecting the spine and thorax, such as scoliosis, pectus excavatum, butterfly vertebrae, and limb abnormalities, including pes cavus, pes planus, club foot, clinodactyly, brachydactyly, and tapered fingers. Gastrointestinal, genitourinary, metabolic, and endocrine systems were less frequently affected (Supplemental Table 1).

**Figure 1:**
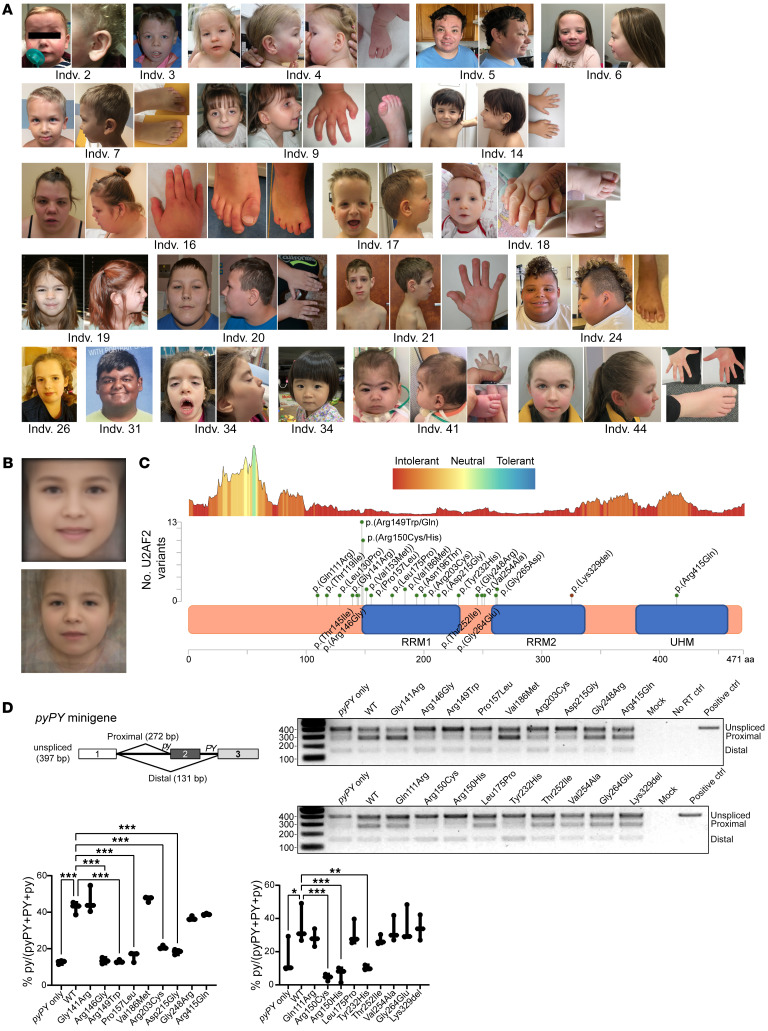
Clinical photographs of affected individuals with *U2AF2* variants demonstrating a shared facial gestalt and molecular analyses demonstrating *U2AF2* variants alter pre-mRNA splicing. (**A**) Individual identifiers correlated with those in Supplemental Table 1. Shared craniofacial features include a prominent/broad forehead, a high anterior hairline, deep-set eyes, short palpebral fissures, downslanting palpebral fissures, a broad nasal root, a narrow nasal bridge with upturned nose, a thin upper lip, micrognathia, a wide mouth, wide-spaced teeth, and a short neck. (**B**) Two average faces generated from controls (top) and all 19 available photos of U2AF2 individuals (bottom) summarizing an identifiable facial gestalt. (**C**) An intolerance landscape plot generated by MetaDome for *U2AF2* variant (NM_001012478.1) analysis (top panel) and a lollipop plot (middle panel) with a schematic outline of the U2AF2 protein domains (lower panel) showing 7 recurrent variants [p.(Arg149Trp), p.(Arg149Gln), p.(Arg150Cys), p.(Val153Met), p.(Arg150His), p.(Val186Met) and p.(Gly265Asp)] and other conserved variants identified in individuals 1–46 and in the DDD study [p.(Pro157Leu) and p.(Thr252Ile)]. (**D**) Eight *U2AF2* variants reduced expression of the longer isoform in the minigene splicing assay, indicative of the pathogenicity of these variants. Normalized ratios are illustrated by the box-and-whisker plot at the bottom (minimum to maximum, showing all the points). *n* = 3 independent experiments. *P < 0.05, **P < 0.01, ***P < 0.001, by ordinary 1-way ANOVA with Dunnett’s multiple comparisons test.

Clinical photographs of 19 participants show several shared craniofacial features, including a prominent/broad forehead, a high anterior hairline, deep-set eyes, hypertelorism, short palpebral fissures, downslanting palpebral fissures, a broad nasal root, a narrow nasal bridge with an upturned nose, thin upper lips, micrognathia, a wide mouth, wide-spaced teeth, and a short neck (Figure 1A), as illustrated in Figure 1B (bottom panel). Moreover, GestaltMatcher (29) analysis revealed that 11 of 19 photos are within a cluster (Supplemental Figure 1A). Notably, 9 of 11 individuals within the cluster carry a variant affecting the residue Arg149 or Arg150, which was also confirmed with the t-distributed stochastic neighbor embedding (*t*-SNE) cluster analysis (Supplemental Figure 1B).

These data reveal a neurodevelopmental phenotype associated with *U2AF2* de novo missense variants, with core features of global developmental, speech, and motor delays, mild-to-severe ID, hypotonia, seizures, frequent brain malformations, autistic features, behavioral disturbances, and a shared facial gestalt.

### De novo U2AF2 variants cluster in RNA recognition motifs.

The *U2AF2* variants (NM_001012478.1) of the 46 probands were identified via exome or genome sequencing and confirmed to be de novo except in 2 instances due to lack of parental DNA. We identified 23 unique variants, of which 22 were missense and 1 was an inframe deletion (Figure 1C). Seven variants [c.445C>T, p.(Arg149Trp), c.446G>A, p.(Arg149Gln), c.448C>T, p.(Arg150Cys), c.449G>A, p.(Arg150His), c.457G>A, p.(Val153Met), c.556G>A, p.(Val186Met) and c.794G>A, p.(Gly265Asp)] were recurrent and were identified in 30 individuals (65% of participants), representing the hotspots of the mutation spectrum (Figure 1C). Of note, the variants c.445C>T, p.(Arg149Trp) and c.448C>T, p.(Arg150Cys) were hyper-recurrent in 11 and 7 individuals (Supplemental Table 2), respectively, who all exhibited the consistent clinical phenotypes (Supplemental Table 1). All identified variants were absent in population genomics resources (i.e., 1000 Genomes Project, ESP6500SI, and gnomAD, version 2.1.2) and in and in-house data set of more than 10,000 exomes. All variants were predicted to be deleterious by multiple bioinformatics prediction algorithms (MutationTaster, Combined Annotation-Dependent Depletion [CADD], Mendelian Clinically Applicable Pathogenicity [M-CAP], etc.) (Supplemental Table 2). All but 1 variant in this cohort clustered within or near 2 critical RNA recognition motifs (RRMs) (Figure 1C and Supplemental Figure 2A) and all variants affecting residues that were predicted to be highly intolerant to variation, as calculated by the MetaDome server (Figure 1C and Supplemental Table 2). Together, we identified 23 *U2AF2* variants associated with an NDD, including 21 de novo and 7 recurrent variants, in the RRMs required for RNA recognition.

### U2AF2 variants impair pre-mRNA splicing.

U2AF2 is an essential pre-mRNA splicing factor for the earliest stage of spliceosome assembly (30). The U2AF2 RRMs serve the major function of recognizing polypyrimidine (Py) tract signals marking 3′ splice sites (31). Considering the locations of the variants in the U2AF2 RRMs, we performed a broader test that included 16 identified and 2 previously described variants (DDD study, ref. 32) distributed across U2AF2 using a well-characterized minigene splicing reporter assay, which consists of 3 exons and 2 intervening Py tracts (uridine-poor [*py*] and uridine-rich [*PY*]) with different splicing efficiencies. We hypothesized that variants occurring physically in close contact with the RNA Py tract would alter the splicing pattern of the *pyPY* transcript. Indeed, we observed that coexpression of 8 of 18 mutant constructs and the *pyPY* minigene in HEK293T cells significantly reduced *py* splicing (Figure 1D), i.e., Arg146Gly, Arg149Trp, Arg150Cys, Arg150His, Pro157Leu, Arg203Cys, Asp215Gly, and Try232His. The abundance of all mutant U2AF2 proteins was comparable to WT (Supplemental Figure 2B and Supplemental Table 2). In addition, RNA-Seq demonstrated that endogenous *U2AF2* expression levels were unchanged in 4 human iPSC lines reprogrammed from lymphoblastoid cells from individuals 1 (Arg149Trp), 4 (Arg149Trp), 6 (Arg150Cys), and 13 (Lys329del), relative to individual 1’s unaffected father and an unrelated control iPSC (|logFC| = 0.19 and *P* = 0.24).

### U2af50 knockdown leads to lethality and defects in neural morphology and function.

**Figure 2:**
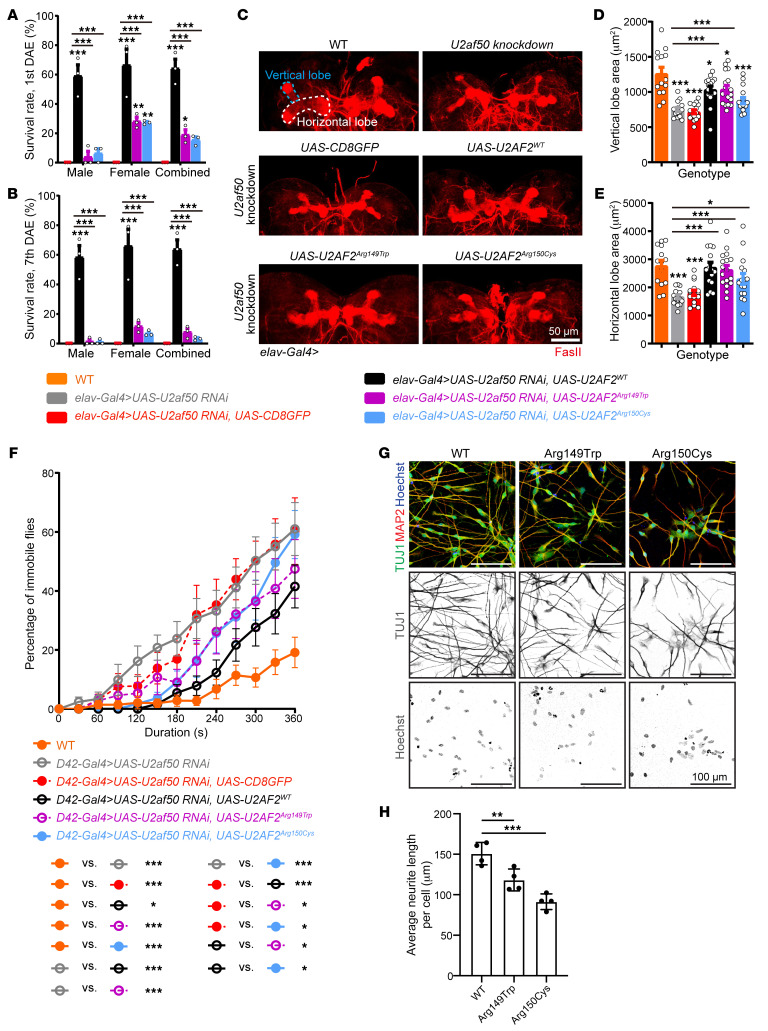
*DrosophilaU2af50*–knockdown, hPSCs, and rescue studies with 2 hyper-recurrent variants. (**A** and **B**) Flies expressing CD8GFP in the setting of *U2af50* knockdown with the *elav-Gal4* driver died before or soon after eclosion. Expression of U2AF2^WT^ drastically increased the survival rate on the first day after eclosion (DAE) (**A**), and almost all the flies were still healthy on the seventh DAE (**B**), whereas variants only slightly increased the survival rate at the first DAE. *n* = 3 groups, with 17–51 flies per group for males and 21–77 flies per group for females. Data indicate the mean ± SEM. *P < 0.05, **P < 0.01, and ***P < 0.001, by 2-way ANOVA followed by Tukey’s test. (**C**–**E**) Pan-neuronal *U2af50* knockdown led to a decrease in the MB area in larvae, whereas U2AF2^WT^ and variants partially (vertical lobe) or fully (horizontal lobe) rescued the phenotype. *n* = 14–17 brains. (**C**) Representative images of MBs. Scale bar: 50 μm. (**D**) Quantification of the vertical lobe area. (**E**) Quantification of the horizontal lobe area. Data indicate the mean ± SEM. *P < 0.05 and ***P < 0.001, by 1-way ANOVA followed by Dunnett’s test. (**F**) *U2af50* knockdown by the *D42-Gal4* driver resulted in increased heat-induced paralysis. Expressing U2AF2^WT^ and variants in *U2af50* knockdown partially attenuated the phenotype. *n* = 6–13 groups, with 7–14 flies per group. Data indicate the mean ± SEM. *P < 0.05 and ***P < 0.001, by 2-way ANOVA followed by Tukey’s test. (**G**) Representative immunofluorescence images of induced neurons on day 2. Scale bars: 100 μm. (**H**) Quantification of the average neurite length per cell normalized by the Hoechst-labeled cell number. Graphs show the mean ± SD of 4 biological replicates. **P < 0.01 and ***P < 0.001, by ordinary 1-way ANOVA with Dunnett’s multiple-comparison test.

Because all of the U2AF2 patients have neurologic deficits and *U2AF2* is highly expressed in the prenatal and postnatal brain (33), we used an in vivo system to study the physiological significance of the variants. For this, we studied *U2af50*, the *Drosophila* ortholog of *U2AF2,* by assessing the functional effects of *U2af50* knockdown. We first expressed *U2af50 RNAiBL27542* under the control of *elav-Gal4*, a pan-neural driver and found that most offspring died before the end of the pupal stage, with very few surviving to adulthood, but all died shortly after eclosion (Figure 2, A and B). We confirmed the lethality in neural *U2af50*–knockdown flies by crossing *elav-Gal4* with 2 other independent RNAi strains, *U2af50 RNAiv24176* and *U2af50 RNAiBL55153*. Compared with WT controls, neural *U2af50* knockdown using the *elav-Gal4* driver led to a 49% reduction in *U2af50* expression based on RNA-Seq data (|logFC| = 0.98 and *P_adj_* = 1.34 × 10^–46^). Although the body lengths were comparable between WT and neural *U2af50–*knockdown larvae (Supplemental Figure 3, A and B), we observed a modest decrease in the larval brain area after *U2af50* knockdown (Supplemental Figure 3, C and D). To further dissect whether cell mitosis was responsible for the reduced brain area, we performed immunostaining with the phosphorylated histone H3 (p-H3) antibody, a mitotic marker (34). We observed that the number of p-H3–positive cells in the brain lobes was significantly decreased in *U2af50 RNAi*–expressing larvae relative to controls (Supplemental Figure 3, C and E). Moreover, in *U2af50*-knockdown brains, the mushroom body (MB), which is the primary learning and memory center, exhibited severe structural defects. Compared with the WT brains, knocking down *U2af50* led to an approximately 40% reduction in both vertical and horizontal lobes of the MB (Figure 2, C–E). To determine if *U2af50* knockdown directly affected MB development, or if the decrease was secondary to generalized brain malformations, we induced expression of *U2af50 RNAi* under the control of *OK107-Gal4*, a MB-specific driver (35). Unlike pan-neural knockdown, knocking down *U2af50* specifically in the MB was not lethal, and in the larval brain the MB showed mild morphological deficits (Supplemental Figure 3, F–H), suggesting that U2af50 was critical for MB development via mitotic and potentially postmitotic mechanisms. To investigate whether the MB-specific knockdown had an effect on fly behavior, we next subjected the mushroom-specific *U2af50*-knockdown virgin females to the social space assay (36) and found that, while WT flies were dispersed throughout the triangle arena, the *U2af50*-knockdown flies were clustered together and much closer to their nearest neighbor (Supplemental Figure 3, I–K), suggesting disrupted social functioning of adult MB neurons as an impairment in learning and memory, which corroborated with some of the behavioral features observed in patients.

As noted above, 23 individuals had childhood febrile seizures and/or generalized tonic clonic seizures accompanied by motor delay. To test whether knocking down *U2af50* in motor neurons is sufficient to trigger seizure in flies, we crossed the *U2af50* RNAi fly stock with *D42-Gal4*, a motor neuron driver (37). The motor neuron *U2af50*-knockdown flies were viable and displayed normal motor performance in the negative geotaxis assay (a climbing test), suggesting that their overall locomotor function was not attenuated (Supplemental Figure 3L). We next bathed the fly vials in hot water and recorded the heat-induced paralysis every 30 seconds up to 6 minutes. All flies behaved normally at the onset of the assay but gradually fell to the bottom of the tube, lying on their back with legs twitching. Interestingly, *U2af50*-knockdown flies showed a substantially shorter latency and higher percentage of paralysis. At 6 minutes, 60% of *U2af50*-knockdown flies were immobile, whereas only 20% of the WT flies showed the heat-induced paralysis (Figure 2F), paralleling the frequent seizures seen in patients (56%). Taken together, these phenotypes observed suggest that *U2af50* played an essential role in the *Drosophila* nervous system, and, most important, they mimic some key brain malformations observed in patients carrying *U2AF2* variants.

### U2AF2 variants are pathogenic and exert partial LoF.

To investigate the functional impact of the identified *U2AF2* missense variants, we generated transgenic flies with inducible expression of 2 recurrent variants that were found in 11 (U2AF2^Arg149Trp^) and 7 (U2AF2^Arg150Cys^) individuals, respectively. We first examined whether U2AF2^WT^ and its variants could rescue the lethality phenotype in neural-specific *U2af50*-knockdown flies. We crossed *UAS-U2AF50^WT^ UAS-U2af50 RNAi/TM6B* with *elav-Gal4 UAS-Dcr-2* flies. In the adult offspring expressing U2AF2^WT^, 60% also expressed *U2af50* RNAi (the remaining flies express TM6B instead), and these flies survived beyond 7 days after eclosion, compared with the dose-controlled RNAi that showed minimal survival (Figure 2, A and B). These data demonstrated that the expression of U2AF2^WT^ dramatically increased the survival rate of *U2af50*-knockdown flies. In contrast, expressing either U2AF2^Arg149Trp^ or U2AF2^Arg150Cys^ only slightly elevated the survival rate of both males and females (Figure 2, A and B). Second, we asked whether the expression of WT or U2AF2 variants could rescue the morphological defects in pan-neural *U2af50*–knockdown brains. We found that the expression of U2AF2^WT^ as well as U2AF2^Arg149Trp^ or U2AF2^Arg150Cys^ could fully rescue the proliferation deficit induced by *U2af50* RNAi (Supplemental Figure 3, C and E). Moreover, U2AF2^WT^ or U2AF2^Arg149Trp^ not only fully rescued the larval brain hemisphere area (Supplemental Figure 3D), but also restored the horizontal lobe area of the MB (Figure 2E). In addition, the vertical lobe area was also partially rescued (Figure 2D). Comparatively, expression of U2AF2^Arg150Cys^ did not rescue the brain area, but modestly rescued the area of both horizontal and vertical lobes (Figure 2, C–E). Third, in the heat-induced seizure paradigm, U2AF2^WT^ in the setting of *U2af50*-knockdown remarkably reduced the percentage of immobile flies, while the 2 variants only partially rescued the phenotype (Figure 2F). These results strongly suggest that, while WT *U2AF2* could largely replace *U2af50* in flies, the 2 variants were partial LoF alleles. It is worth noting that knocking down *U2af50* in fly larval sensory neurons using *ppk-Gal4* (38) resulted in partially thinned/fragmented/degenerative dendrites that could be fully rescued by human WT U2AF2 and variants (Supplemental Figure 3M), suggesting the diverse function of U2AF2/U2af50 in neural circuit assembly and its indispensable role in postmitotic neurons.

### Neurite growth defect in knockin isogenic hPSCs.

To unveil the effect of patient variants in cortical neuron differentiation, we introduced the U2AF2^Arg149Trp^ or U2AF2^Arg150Cys^ hyper-recurrent variants into H9 hPSCs using CRISPR/Cas9-based genome editing to generate an isogenic model. The knockin was confirmed by Sanger sequencing and RNA-Seq with the heterozygous state of patient variants and synonymous variants that prevent recutting with Cas9 (Supplemental Figure 4A). After validation of genome integrity by genotyping and preservation of pluripotency markers by RNA-Seq, knockin hPSCs and WT cells (with synonymous variants) were further differentiated using the NGN2-mediated neuronal induction approach (39). We started to observe neurite outgrowth from both WT and 2 mutant clones within 48 hours. Compared with WT cells, neurons differentiated from mutant hPSCs demonstrated decreased neurite length (Figure 2, G and H). We confirmed that the neurite growth deficit was most likely not caused by protein mislocalization, as the variants did not change the localization of U2AF2 in the nucleus (Supplemental Figure 4B), or by a general defect in proliferation (Figure 2G). Together, these data suggest that U2AF2 has an important role in neurogenesis and neuritogenesis and a partial LoF effect for both variants, which would ultimately alter neuronal connectivity and network activity.

### PRPF19 variants are associated with NDD and autism.

**Figure 3:**
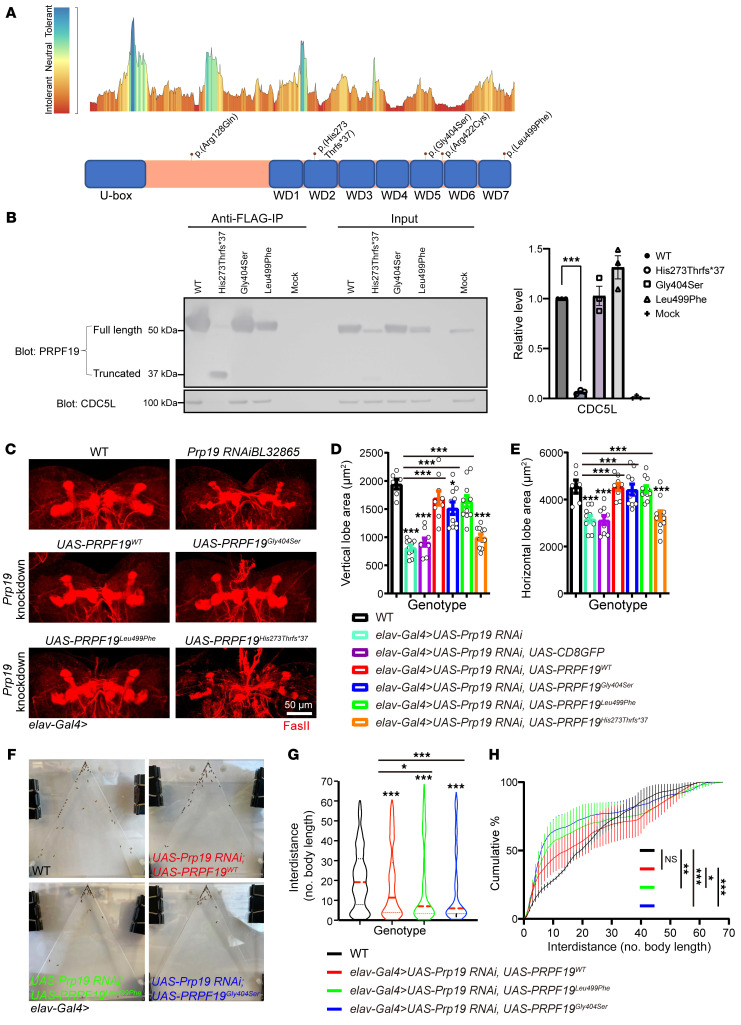
Modeling PRPF19 in in vitro cellular and in vivo fly models. (**A**) An intolerance landscape plot generated by MetaDome for *PRPF19* variant (NM_014502.5) analysis (top panel) and a schematic outline of the PRPF19 protein domains (lower panel). (**B**) Western blot and co-immunoprecipitation analysis of overexpressed PPRF19^WT^, PRPF19^Gly404Ser^, PPRF19^Leu499Phe^, and PPRF19^His273Thrfs*37^with FLAG tag. Graph shows the mean ± SEM. *n* = 3 independent experiments. ***P < 0.001, by 2-tailed, paired *t* test. (**C**–**E**) Pan-neural *Prp19* knockdown led to reduced MB area. Expression of human PRPF19^WT^, PRPF19^Gly404Ser^, or PPRF19^Leu499Phe^ fully or partially rescued the area, while PPRF19^His273Thrfs*37^ failed to attenuate the structural defects. *n* = 6–11 brains. (**C**) Representative images. Scale bar: 50 μm. (**D**) Quantification of the vertical lobe area. (**E**) Quantification of the horizontal lobe area. Data indicate the mean ± SEM. *P < 0.05 and ***P < 0.001, by 1-way ANOVA (**D** and **E**). (**F**–**H**) In the social behavior assay, flies expressing the 2 *PRPF19* missense variants in the setting of *Prp19* knockdown displayed disrupted social behavior as revealed by their aberrant distribution in the device and reduced interdistance. In comparison, expression of PRPF19^WT^ in the setting of *Prp19* knockdown partially restored the impaired social behavior. *n* = 3 groups, with 26–42 flies per group. (**F**) Representative images showing fly distribution in the social behavior assay. (**G**) Quantification of the distance between any of the 2 flies in 1 trial. *P < 0.05 and ***P < 0.001, by 2-tailed, unpaired *t* test. (**H**) Cumulative frequency of fly interdistance in the device.*P < 0.05, **P < 0.01, and ***P < 0.001, by 2-way ANOVA followed by Šidák’s test. .

The spliceosomal complex has more than 200 proteins (40), but only a few have been associated with NDDs or neurodegenerative disorders (19–22). Consequently, we considered genes involved in the splicing process as candidates for NDD and reanalyzed research or clinical sequencing data of a cohort of 245 unresolved NDD cases. In 1 previously negative clinical exome, we identified a de novo variant, c.1210G>A, p.(Gly404Ser), in *PRPF19* (P1; NM_014502.5; OMIM 608330). By GeneMatcher, we identified 5 more patients with heterozygous de novo *PRPF19* variants: c.1495C>T, p.(Leu499Phe) (P2), a 400 kb duplication spanning *PRPF19* (P3), c.1264C>T, p.(Arg422Cys) (P4), c.383G>A, p.(Arg128Gln) (P5), and c.816delT, p.(His273Thrfs*37) (P6) (Figure 3A and Supplemental Table 3). Individuals P1–P5 all have speech/language and motor delays, and individuals P1–P4 have a formal diagnosis of autism. Moreover, hypotonia was present in 3 individuals (P1, P2, and P4). Three individuals (P1, P4, and P5) had ID and 1 was normal (P3). One individual was too young for ID assessment (P2), and P6 was a fetus with multiple congenital malformations (Table 1 and Supplemental Table 3), substantially overlapping the core features of *U2AF2*-related NDD. All variants were confirmed by Sanger sequencing and were absent in population genomics resources. Prediction of their deleterious effects is summarized in Supplemental Table 2. *PRPF19* is intolerant to both LoF (probability of LoF intolerance [pLI] = 1) and missense (*Z* = 3.89) variants according to the gnomAD population database.

*PRPF19* encodes pre-mRNA processing factor 19, which functions in splicing and more specifically in the catalytic activation and structural stabilization of the spliceosome (41–43). The Prp19 complex, also known as NTC, has PRPF19 as one of the core subunits (i.e., PRPF19, CDC5L, and BCAS2) assembling the other Prp19 complex–associated proteins via C-terminal WD40 domains (44–47). Spliceosome assembly and activation is a dynamic process: the U1 and U2 snRNPs (including U2AF2) mark an intron boundary, then the pre-mRNA binds to U4/U6-U5 tri-snRNP, followed by the release of both U1 and U4 with the arrival of the Prp19 complex to activate the spliceosome (15). However, some reports suggested that the Prp19 complex is recruited to the early spliceosome independent of the tri-snRNP, and that U2AF2 may be involved in the process (48). The 2 missense *PRPF19* variants (Gly404Ser and Arg422Cys) are in the WD40 beta-propeller architecture 5 and 1 (Leu499Phe) in WD40 beta-propeller architecture 7 (Figure 3A). 3D structural modeling (Supplemental Figure 2A, PDB: 4LG8) showed that these substitutions, whose side chains are bigger or smaller than WT residues (49), are expected to destabilize the beta-propeller folding and in turn impair association with NTC-associated proteins. We induced expression of the WT and 3 PRPF19 variants in HEK293T cells, and, as expected, cells transfected with PRPF19^His273Thrfs*37^ showed a complete loss of binding with CDC5L (Figure 3B), suggesting that the variant p.(His273Thrfs*37) is a complete LoF allele.

### PRPF19 pathogenic variants lead to social behavior deficits.

To dissect the pathophysiological mechanism of how *PRPF19* variants contribute to disease, we analyzed fly ortholog *Prp19* with 2 independent RNAi lines (*Prp19 RNAiBL32865* and *Prp19 RNAiv41438*). RNA-Seq analysis showed a knockdown efficiency of 77% (|logFC| = 2.09 and *P_adj_* = 1.56 × 10^–15^). As expected, all pan-neural *Prp19*–knockdown flies died before eclosion (Supplemental Figure 5A) and resulted in a generalized reduction in brain cell proliferation (Supplemental Figure 5B). Of note, overexpression of PRPF19^WT^ significantly increased cell proliferation (Supplemental Figure 5B), consistent with the phenotype observed in participant P3, who has an increased HC (97%; *Z* = 1.95) and a tall stature. Since individuals carrying *PRPF19* missense variants have motor deficits, we next examined the locomotor function in pan-neural *Prp19* RNAi larvae in the open-field locomotion assay. We assessed the total crawling length, distance, and speed and found that RNAi larvae had overt hypoactivity (Supplemental Figure 5C). Pan-neural *Prp19* RNAi also led to structural morphology defects in the MB at the larval stage (Figure 3C). Similar to the phenotype caused by *U2af50* knockdown, both the vertical and horizontal lobes of the MB exhibited remarkable area reduction in *Prp19* RNAi larvae (Figure 3, D and E). The expression of PPRF19^WT^ or either of the 2 missense variants (PRPF19^Gly404Ser^ and PPRF19^Leu499Phe^) could fully or partially rescue the structural defect in the MB and survival rate, with no significant difference in the rescue efficacy of the missense variants compared with WT. However, the truncated form of human PRPF19 [p.(His273Thrfs*37)] failed to rescue these phenotypes (Figure 3, C–E, and Supplemental Figure 5A), corroborating the fact that the variant c.816delT, p.(His273Thrfs*37) is indeed a complete LoF allele. Moreover, compared with PRPF19^WT^ and PPRF19^Leu499Phe^, which fully rescued the proliferation deficit, PRPF19^Gly404Ser^ had reduced rescuing of larval brain proliferation (Supplemental Figure 5B). Nevertheless, PRPF19^WT^ and both PRPF19^Gly404Ser^ and PPRF19^Leu499Phe^ variants fully rescued the lethality in *Prp19* pan-neural knockdown flies (Supplemental Figure 5A), allowing us to conduct the social space assay to further test the effect of these 2 de novo missense variants on social behavior related to the autism diagnosis in patients. We found that female flies expressing either PRPF19 variant exhibited a reduction in social space as determined by the decreased interdistance when compared with flies expressing PRPF19^WT^, which were similar to WT flies (Figure 3, F–H). This result indicated that, although the 2 *PRPF19* missense variants drastically extended the lifespan of *Prp19*-knockdown flies, they did not completely rescue their social behaviors. Thus, Prp19 was required for normal social behaviors of flies, and de novo *PRPF19* variants affected neurons regulating social functions and most likely caused neurophysiological defects.

### Dysregulation of transcripts associated with neurodevelopmental processes.

Since U2AF2 and PRPF19 have converging functions in splicing and variants of both genes cause overlapping NDDs with similar features, we hypothesized that there may be common downstream effectors regulated by these genes. To test this hypothesis, we performed transcriptome profiling of brain tissues harvested at the third instar larval stage from WT controls and *elav-Gal4* driving knockdown flies for *U2af50* and *Prp19*. In *U2af50* RNAi fly brains, we identified 1,219 differentially expressed genes (DEGs) (|logFC| ≥ 0.7 and *P_adj_* ≤ 0.01; Supplemental Figure 6, A and B), of which 70.6% were upregulated (*n* = 861) and 29.4% were downregulated (*n* = 358). DEGs were subsequently analyzed through the DAVID Functional Annotation Resource, and we found significant enrichment of downregulated DEGs in 9 Gene Ontology (GO) terms, which included axon guidance, negative regulation of neuroblast proliferation, neuron differentiation, and others (Supplemental Figure 6C). Similarly, we identified 1,498 DEGs in *Prp19* RNAi, with 1,085 being upregulated and 413 downregulated. Enrichment analysis similarly revealed 14 significant GO terms (|logFC| ≥ 1 and *P_adj_* ≤ 0.01; Supplemental Figure 6, D and E), including neuron differentiation, regulation of neurogenesis, regulation of neuroblast proliferation, and axon guidance (Supplemental Figure 6F). These results suggest that both genes play essential roles in neurodevelopmental processes, which could be mapped to fly MB development and patient phenotypes, especially brain function and neurodevelopment.

### Shared splicing alteration by U2af50 and Prp19 knockdown.

**Figure 4:**
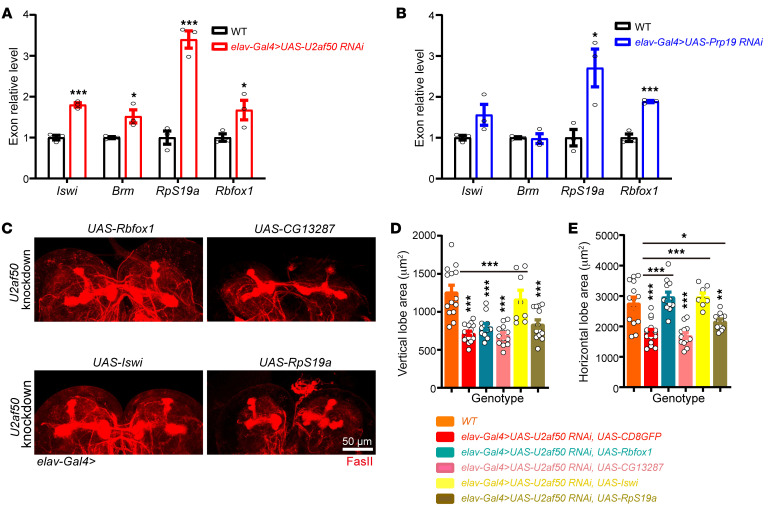
RT-qPCR confirms potential substrates or downstream effectors that robustly rescue MB defects in *U2af50-*knockdown brains. (**A** and **B**) RT-qPCR data confirmed 4 exon candidates from fly brain RNA-Seq and show that Iswi, Rbfox1, and RpS19a might be the shared downstream splicing targets of U2af50 and Prp19. *n* = 3. (**A**) Compared with WT, increased exon inclusions in Iswi, RpS19a, and Rbfox1 were significantly upregulated in *U2af50*-knockdown brains. *P < 0.05 and ***P < 0.001, by 2-tailed, unpaired *t* test. (**B**) The expression level of the same differentially used exons in Iswi, RpS19a, and Rbfox1 was also upregulated in *Prp19*-knockdown brains. Data indicate the mean ± SEM. *P < 0.05 and ***P < 0.001, by 2-tailed, unpaired *t* test. (**C**–**E**) The expression of Rbfox1, Iswi, or RpS19a fully or partially rescues the decreased MB area in *U2af50*-knockdown brains. *n* = 8–16. (**C**) Representative images. Scale bar: 50 μm. (**D**) Quantification of the vertical lobe area. ***P < 0.001, by 1-way ANOVA followed by Dunnett’s multiple-comparison test. (**E**) Quantification of the horizontal lobe area. Data indicate the mean ± SEM. *P < 0.05, **P < 0.01, ***P < 0.001, by 1-way ANOVA followed by Dunnett’s multiple-comparison test.

To further probe the involvement of the 2 genes in mRNA splicing and prioritize potential common downstream targets, we analyzed differentially used exons (diffUEs) by 3 algorithms, DEXSeq (50), limma (51), and JunctionSeq (52), and retained candidates supported by at least 2 algorithms. Analysis of *U2af50* RNAi RNA-Seq data with moderate knockdown efficiency (49%) revealed 234 diffUEs in 185 unique genes (Supplemental Table 4), and *Prp19* RNAi (77% knockdown efficiency) led to 1,090 diffUEs in 727 unique genes (Supplemental Table 5). The numbers of affected exons are consistent with the different knockdown efficiencies and also with the respective functions of U2af50 and Prp19 orthologs in splice site selectivity (31) and general spliceosome activation (53). Gene set enrichment analysis of these 727 genes similarly demonstrated that the significant gene categories hit many neurodevelopmental processes, such as axon guidance, dendrite morphogenesis, MB development, and axonogenesis (Supplemental Figure 6G). We intersected significant diffUEs across the 2 data sets and identified 117 shared diffUEs. Four exon candidates with increased inclusion were then selected for real-time RT-qPCR validation, due to the implication of their human orthologous genes in human rare disorders. These exons belong to the genes *Iswi*, *Brm*, *RpS19a*, and *Rbfox1*, and increased inclusion of all 4 and 3 exons within each gene were confirmed in *U2af50* and Prp19 RNAi, respectively (Figure 4, A and B), suggesting that they are potential substrates of the spliceosome.

We hypothesized that increased inclusions of noncanonical exons (i.e., alternative first exons) decrease the expression of canonical transcript(s), and compensating the diminished expression of the canonical isoform in fly pan-neurons could alleviate the MB defects. Remarkably, 3 genes tested, *Iswi*, *Rbfox1*, and *RpS19a*, could rescue the MB defects observed in flies expressing *U2af50* RNAi to a different extent. Expressing Iswi, Rbfox1, or RpS19a in the setting of *U2af50* knockdown significantly increased the area of the horizontal lobe of the MB. Strikingly, expression of Iswi in *U2af50* RNAi fully restored the area of both vertical and horizontal lobes (Figure 4, C–E). However, Iswi and Rbfox1 failed to rescue the mushroom defects in *Prp19*-knockdown flies (Supplemental Figure 7). These results demonstrate both hierarchy and specificity in U2AF2 and PRPF19 substrates or downstream effectors, depicting the complexity of the genetic network mediating neural development.

### Mis-splicing events further revealed by hPSC RNA-Seq.

To isolate the effect of a single variant change, we performed transcriptomic profiling of the hPSC isogenic model with 24 samples (10 colonies of Arg149Trp, 4 colonies of Arg150Cys, and 10 WT colonies; Supplemental Figure 6H). Interestingly, we found 62 diffUE events (|log_2_FC|>0.7 and *P_adj_* ≤ 0.01) in 39 unique genes (Supplemental Table 6 and Supplemental Figure 6, I–K). Gene enrichment analysis of these 39 genes revealed that only RNA binding under molecular functions was enriched (*P_adj_* = 0.017). Real-time reverse transcription quantitative PCR (RT-qPCR) confirmed 3 randomly selected exon targets, i.e., *RPL37*, *DNAJC21*, and *OTX2* (Supplemental Figure 6, I–K). These data suggest that the 2 pathogenic variants (Arg149Trp and Arg150Cys) residing in the interaction surface with RNA could result in aberrant mRNA splicing in stem cells, a relevant cell type to model the pathophysiology.

### Missense variants in RBFOX1 associated with a mild NDD.

**Figure 5:**
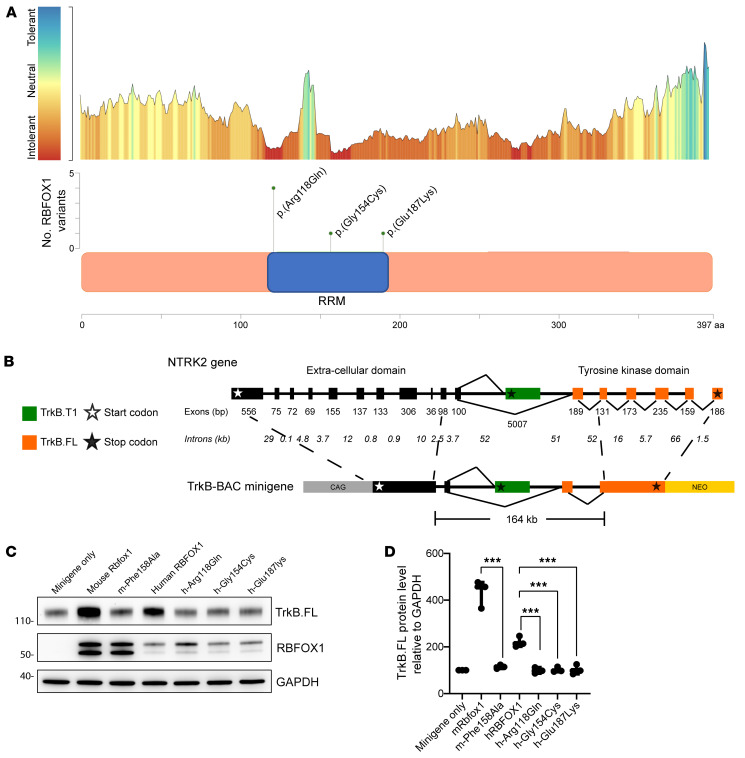
The identified *RBFOX1* missense variants alter the gene-splicing pattern. (**A**) Intolerance landscape plot generated by MetaDome for *RBFOX1* (NM_018723.4) variant analysis (top panel) and a lollipop plot (middle panel) with a schematic outline of the RBFOX1 protein domains (lower panel) showing 1 recurrent variant, p.(Arg118Gln), and other conserved variants identified in individuals 5 and 6. (**B**) Schematic representation of the murine TrkB gene (top) and the TrkB-BAC minigene (bottom). The exons in black are commonly expressed in both the full-length (TrkB.FL) and the truncated T1 (TrkB.T1) isoforms. The TrkB.T1 isoform was generated by including the specific T1 exon (green), whereas the TrkB.FL isoform was generated by including exons in orange. The 164 kb TrkB-BAC minigene includes upstream, the transmembrane coding exon and, downstream, in addition to the TrkB.T1 coding exon, the 2 exons encoding the juxtamembrane region preceding the tyrosine kinase region. The cDNAs coding for the missing extracellular domain and the tyrosine kinase region of TrkB were fused inframe to an upstream exon (98 bp) and a downstream exon (131 bp) of the BAC region (dashed lines). A neomycin resistance cassette (NEO) is present in the minigene for selection, while a synthetic CAG promoter drives the minigene expression. (**C**) Representative immunoblot analysis of the clonal cell line expressing the TrkB-BAC minigene transiently transfected with plasmid vectors expressing the mouse Rbfox1 and the human RBFOX1 (as positive controls), the F158A mutant (as a negative control), and the de novo RBFOX1 variants. Ntrk2 (TrkB) protein levels were tested 48 hours after transfection with an antibody against the TrkB intracellular domain to specifically detect the full-length protein (TrkB.FL). (**D**) Immunoblot quantification analysis of TrkB.FL protein levels from 3 independent experiments, as in **C**. *n* = 3. Data indicate the mean ± SEM. ***P < 0.001, by ordinary 1-way ANOVA with Dunnett’s multiple-comparison test.

Guided by these functional data, and in particular our results showing that RBFOX1 acted as a potential substrate and downstream effector of spliceosome malfunction, reanalysis of clinical negative exome data indeed revealed a de novo variant (c.353G>A, p.Arg118Gln) in *RBFOX1* (NM_018723.4; OMIM 605104) in a patient with mild global developmental delay, hypotonia, ataxic gait, delayed myelination, and brain frontal volume loss, overlapping with the clinical symptoms observed in patients with *U2AF2* variants. Through GeneMatcher, we recruited 5 additional patients. All 6 patients presented with mild neurological symptoms ranging from normal intelligence and a mild DD affecting verbal and motor skills on the mild end of the spectrum to mild-to-moderate ID, autism spectrum disorder, and seizures on the severe end of the spectrum (Table 1 and Supplemental Table 7) . All *RBFOX1* variants were confirmed to be de novo, with the p.(Arg118Gln) variant being recurrent in 4 unrelated individuals (Figure 5A), and other 2 individuals having the c.460G>T, p.(Gly154Cys) and c.559G>A, p.(Glu187Lys) variant. RBFOX1 is not directly involved in the core spliceosome complexes but has a single RRM domain that binds to specific sequences [5′-(U)GCAUG-3′] to promote inclusion or exclusion of exons (24, 25) and is expressed mainly in the nervous system, heart, and muscle (25, 54). All identified missense variants occurred in the only RRM domain, and multiple algorithms predicted potential deleterious effects (Supplemental Table 2). Moreover, inspection of the protein structure (Supplemental Figure 2A, PDB: 2N82) demonstrated that all 3 missense variants are in a region of close contact with RNA, as for many *U2AF2* variants, suggesting that the RNA-binding affinity may be impaired.

To functionally test the pathogenicity of these variants, we utilized an in vitro minigene system sensing the alternative splicing activity of Rbfox1. Since expression of 2 TrkB isoforms by the TrkB locus is regulated by Rbfox1 (55), we generated a 164 kb bacterial artificial chromosome (BAC) containing part of the mouse TrkB locus including the alternatively spliced exons (Figure 5B). Cotransfection of the minigene and either the murine Rbfox1 or the human RBFOX1 in HEK293T cells caused a significant upregulation in the levels of the TrkB full-length (TrkB.FL) specific isoform (Figure 5C). As expected (25), a known mouse Rbfox1 pathogenic variant (F158A) did not change TrkB.FL expression levels (Figure 5C). Interestingly, RBFOX1^Arg118Gln^, RBFOX1^Gly154Cys^, and RBFOX1^Glu187Lys^ expressed at a level similar to that in the human WT construct did not modulate TrkB.FL isoform expression (Figure 5, C and D). Altogether, these data suggest that these missense variants uncovered in the RRM domain of RBFOX1 impaired its splicing function, and these pathogenic de novo missense variants in the RRM domain were associated with a mild NDD (Supplemental Table 2).

## Discussion

We describe 3 NDDs caused by de novo heterozygous variants in *U2AF2*, *PRPF19*, and *RBFOX1*, all of which are involved in the pre-mRNA splicing machinery. *U2AF2* and *PRPF19* encode core spliceosome subunits to guide the early stage of splice-site choice and spliceosome activation, respectively. *RBFOX1* encodes a tissue-specific splicing factor that regulates splicing through binding to the pre-mRNA *cis*-acting element 5′-(U)GCAUG-3′. Variants in *U2AF2* and *RBFOX1* clustered predominantly in RNA recognition motifs, while a majority of *PRPF19* variants were in the WD40 domain. Remarkably, all of the patients converge on similar neurodevelopmental phenotypes (Table 1), including mild-to-severe developmental delays, intellectual disability, hypotonia, seizures, and autism.

A group of rare Mendelian diseases caused by pathogenic variants in genes encoding core spliceosomal complexes have been characterized as spliceosomopathies, e.g., SF3B4 involved in the U2 snRNP in Nager syndrome, an acrofacial dysostosis; SNRPB of the U1/2/5/4/6 snRNP in cerebrocostomandibular syndrome, characterized by Robin sequence and rib defects; and U4/U6 snRNP subunits (PRPF3, PRPF4, and PRPF31) and U5 snRNP subunits (PRPF6, PRPF8, and SNRNP200) in retinitis pigmentosa. Most recently, syndromic intellectual disability has been described as an emerging phenotype linking variants in 2 genes, *PUF60* and *FAM50A,* which encode spliceosomal components of the U2 snRNP and the C-stage spliceosome complexes, respectively (22, 56). Our study, by combining human genetics and in vitro molecular and hPSC cellular models, together with the fly in vivo model provides strong evidence supporting a critical role for the spliceosome in neurodevelopment (Supplemental Table 2).

Despite the major role of U2AF2 in pre-mRNA splicing, facilitating the recognition of exon-intron boundaries and identification of the 3′ splice site during the spliceosome assembly, until recently, U2AF2 passenger somatic variants have only been loosely linked to cancer (57). The identification of missense variants in *U2AF2*, coupled with the extensive in vitro and in vivo functional data presented here, has broadened our understanding of its role in neurodevelopment and in splicing biology. It is worth noting that, while this work was in progress, a large NDD cohort study (including the DDD data set) and a few case reports nominated an association between U2AF2 and NDD but with limited clinical information and without functional validation (58–61). It has been suggested that partial RNAi knockdown of the fly *U2af50* results in reduced neuroblasts in the larval brain associated with blunted neuroblast lineages and abnormal neuroblast shape (62). We further demonstrated that 2 hyper-recurrent *U2AF2* variants reduced neuritogenesis in the hPSC isogenic model and resulted in MB patterning defects, seizures, and social deficits in the fly model.

PRPF19 is an indispensable core subunit of the dynamic hPrp19 complex. Knockout of its mouse ortholog has been shown to cause lethality at an early stage of embryonic development (63). We also found that depleting *Drosophila dPrp19* with pan-neural or motor neuron–specific Gal4 caused lethality before eclosion. Even though our PRPF19 sample size was modest (*n* = 5), we discovered 3 types of variants and 2 pathogenic mechanisms besides LoF. The whole gene duplication resulted in elevated proliferation in brain mitotic neurons, which correlated with an NDD phenotype with macrocephaly and tall stature in participant P3. The frameshift variant truncated the WD40 domain, leading to a complete loss of hPrp19 complex formation with CDC5L, which highly suggested that its canonical splicing function may have been lost. In the fly rescue study, the truncated form failed to reinstate normal MB patterning. This may explain why the fetus (P4) with p.(His273Thrfs*37) has the most severe congenital abnormalities (Supplemental Table 3). Absence of any other LoF alleles (including intragenic deletion) in publicly available population genomic data sets and our in-house genomic data suggests that *PRPF19* is an essential gene for human embryonic development, and the fetus P4 is in that sense unique. By contrast, 2 human missense variants could largely replace *dPrp19* and rescue the MB structural defect and adult lethality phenotypes. However, they failed to restore normal social interaction. In addition to the essential role in splicing, PRPF19 could form different complexes that are implicated in the DNA damage response, transcription elongation, mRNA export, and protein degradation (45). Whether other functions of PRPF19 contribute to the phenotypes observed in our study should be further investigated. Nevertheless, by characterizing 4 variants (2 missense, 1 LoF, 1 microduplication) at different cellular levels, we demonstrated the crucial function of PRPF19 in neurodevelopment and provide a promising tool to study the effect of pathogenic variants in vivo. This highlights the finding that our fly model is robust for interrogating not only general neural developmental defects, but also specific and more subtle defects that are spatiotemporally dependent.

It is noteworthy that *PRPF19* missense variants cause clustering or reduce inter-fly distance. Similarly, knockdown of *U2af50* in the MB resulted in increased social aggregation in adult female flies. This suggests that the spliceosome complex is critical for normal development of the MB, which is the center for integrating and processing diverse cues, especially the visual and olfactory inputs that are most important for fly social behavior. Loss of *U2af50* or *Prp19* may lead to compromised MB function in neural processing and in the regulation of higher-order social behavior, mimicking the patients with impaired intellectual functions. Interestingly, different from the *elav-GAL4*>UAS-ISWI RNAi flies, which exhibited increased social space in relation to genetic controls (64), our results resembled what was observed in *Drosophila* neuroligin 4-deficient (dNlg4-deficient) flies (65), which also show an increased tendency to aggregate. This corroborates the wide range of genetic causes for ID or ASD and their spectrum of social deficits.

Our work aims to establish a genetic network associated with NDD, through gene discovery, functional validation, and mechanistic inquiry. We have successfully pinpointed several critical nodes encompassing the U2AF2/PRPF19/RBFOX1 axis. We have revealed hierarchy among the various nodes, and even within the spliceosome complex, with U2AF2 and PRPF19 being closer to the master regulator and RBFOX1 being their substrate and executer. This represents the first layer of the nodes. We have further identified an additional substrate for the spliceosome complex, namely Iswi/SMARCA5/SMARCA1, and downstream effectors such as Rps19a, representing layers 2 and 3 of the nodes. The rescue efficiency of these factors correlates well with their position in the hierarchy. A pressing question is how mutations in a general machinery, such as the spliceosome, lead to relatively specific malfunctions in the nervous system. We would like to postulate at least a few possible scenarios. The expression of the spliceosome complex subunits may be unevenly distributed across tissue types, and there may be enriched expression of U2AF2/PRPF19/RBFOX1 in the brain. There may also be a possible enrichment of the substrates in the nervous system, especially during the critical time window of neurodevelopment. Alternatively, the composition of coregulators or the presence of modulators may differ among cell types. Intriguingly, it was reported that stringent conservation of alternative splicing is present in only a subset of tissues including the brain and is frequently lineage specific (66). Concomitantly, alternative splicing is also the highest in the brain compared with other tissues (67, 68), and is critical for producing neural circuit diversity and specificity. It may be possible that the dysfunction of mutated spliceosome proteins in the brain is less efficiently compensated, given its more stringent configuration. The high energy consumption in the brain might be another potential contributor, which might affect the dynamics, activity, and throughput of the spliceosome, especially considering the fact that the spliceosome is an energy-intensive machinery (69). During brain development, multiple genes have to be expressed in a regulated and coordinated fashion. A defect in spliceosomes will perturb this machinery, leading to a developmental stagnation of the brain’s finer functions. A key step forward will be to systematically dissect more nodes in the network, characterize their spatiotemporal expression, and map out their functional interactions.

In summary, our study implicates the spliceosome in neurodevelopment. It has been widely acknowledged that alternative pre-mRNA splicing is an important mechanism for regulating gene expression for multiple essential genes in the CNS, where it both affects neuronal differentiation and development and controls functions in the mature neurons. The data described in this study advance our understanding of the mechanistic underpinnings of neurodevelopment and neuropsychiatric traits and identify 3 genes to add to the causal gene list in NDDs.

## Methods

### Statistics.

The data shown represent the results of at least 3 separate experiments. All results are presented as the mean ± SEM or the mean ± SD. Comparisons between 2 groups were analyzed by *t* test, and comparisons between 3 groups were done by 1-way ANOVA followed by Dunnett’s test or 2-way ANOVA followed by Turkey’s or Šidák’s test using GraphPad Prism (GraphPad Software). A *P* value of less than 0.05 was considered statistically significant.

### Study approval.

Informed consent was obtained from all the families according to protocols approved by local IRBs and human research ethics committees. Specific written consent was also retained for all individuals whose photographs appear in the manuscript.

### Data availability.

RNA-Seq data generated from flies and hPSCs are available in the NCBI’s Gene Expression Omnibus (GEO) database (GEO GSE246137). Supporting data for all values underlying the data presented in the graphs are provided in the Supplemental Supporting Data Values file.

## Author contributions

DL, YS, and HH conceptualized the study. DL, AB, DGMB, GVH, LG, AKP, LAP, GA, MH, SB, MS, RKP, ZS, EHZ, BAK, EJB, MHH, GL, KMB, M Tarnopolsky, ML, M Towne, LB, MJP, HF, LKP, EA, MLD, AN, MW, RP, KL, MS, PS, MI, FZ, AC, TJM, MB, RRL, TBB, RL, ML, JD, CN, AA, JH, BK, SMM, MMM, JAM, AMR, EMM, MJF, SMR, IH, RSM, MG, BDG, VJ, AW, JG, SJ, KJ, AB, CQ, VKM, EC, CR, DG, AA, MM, VN, RS, YU, NM, RT, KT, YS, GY, BW, MW, BK, ACEH, MLT, RS, LR, RCS, VS, EJH, DC, AC, GC, TK, BF, FTM, LFGM, SG, MO, LC, NQ, DC, CL, AS, MK, MN, BOJ, PS, KS, JA, VP, SM, MV, CP, CF, KF, RKH, SB, JR, CS, MC, CB, JG, KL, AS, CM, RP, BBADV, MAVS, ASB, BC, TR, ZT, and HH conducted molecular and clinical evaluation of the affected individuals. DL, QW, MRB, YZ, AA, MEM, CH, LT, FT, MEP, NA, and YS performed functional assessment and validation. DL and NO were responsible for project administration. TH and PMK conducted the GestaltMatcher analysis. DL, QW, and YS wrote the original draft of the manuscript. All authors reviewed and edited the manuscript. DL, YS, and HH supervised the study. The order of co–first authors was determined on the basis of their contributions to the study and drafting of the manuscript, and a consensus agreement was reached to ensure mutual understanding and commitment.

## Supplementary Material

Supplemental data

Supplemental table 1

Supplemental table 2

Supplemental table 3

Supplemental table 4

Supplemental table 5

Supplemental table 6

Supplemental table 7

Supplemental table 8

Supporting data values

## Figures and Tables

**Table 1:**
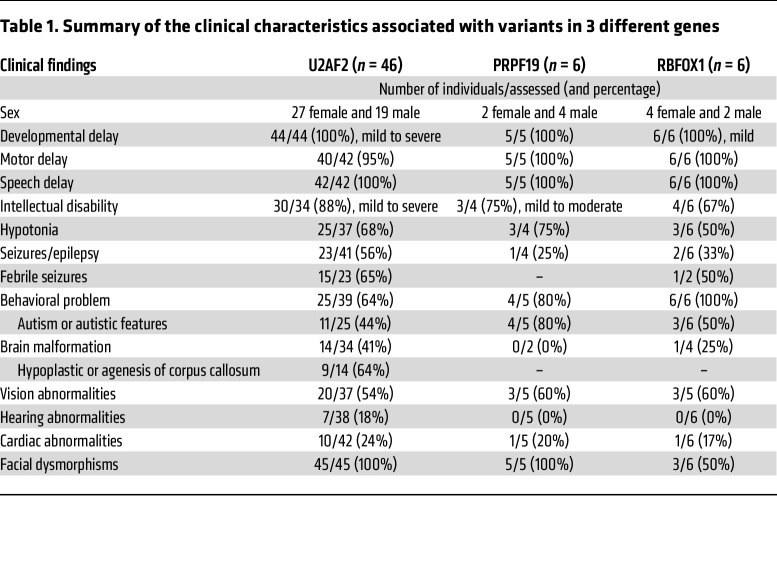
Summary of the clinical characteristics associated with variants in 3 different genes

## Version 1 11/14/2023

In-Press Preview

## Version 2 01/02/2024

Electronic publication
